# Ultrasound in the evaluation of enthesitis: status and perspectives

**DOI:** 10.1186/ar3516

**Published:** 2011-11-17

**Authors:** Frédérique Gandjbakhch, Lene Terslev, Fredrick Joshua, Richard J Wakefield, Esperanza Naredo, Maria Antonietta D'Agostino

**Affiliations:** 1 Rheumatology Department, Université Paris 6-Pierre et Marie Curie, Hôpital La Pitié Salpetrière, APHP, 83 Boulevard de l'hôpital 75013 Paris, France; 2 Rheumatology Department, Copenhagen University Hospital at Glostrup, Nordre Ringvej 57 2600 Glostrup, Denmark; 3 Rheumatology Department, Prince of Wales Hospital, Barker St Randwick NSW 2031 Australia; 4 Section of Musculoskeletal Disease, LIMM, University of Leeds and NIHR Leeds Musculoskeletal Biomedical Research, Chapeltown Road Leeds LS7 4SA,UK; 5 Rheumatology Department, Hospital Universitario Severo Ochoa; Doctor Alvarez Sierra 4, 4° A, 28033 Madrid, Spain; 6 Rheumatology Department, Université Paris Ouest-Versailles-Saint Quentin en Yvelines, Hôpital Ambroise Paré, APHP, UPRES EA 2506, 9 avenue Charles De Gaulle 92100 Boulogne-Billancourt, France

**Keywords:** Systematic literature review, scoring system, ultrasound, power Doppler, enthesitis, enthesopathy, spondyloarthritis, ankylosing spondylitis, OMERACT filter

## Abstract

**Introduction:**

An increasing number of studies have applied ultrasound to the evaluation of entheses in spondyloarthritis patients. However, no clear agreement exists on the definition of enthesitis, on the number and choice of entheses to examine and on ultrasound technique, which may all affect the results of the examination. The objectives of this study were to first determine the level of homogeneity in the ultrasound definitions for the principal lesions of enthesitis in the published literature and second, to evaluate the metric properties of ultrasound for detecting enthesitis according to the OMERACT filter.

**Methods:**

Search was performed in PUBMED and EMBASE. Both grey-scale and Doppler definitions of enthesitis, including describing features of enthesitis, were collected and metrological qualities of studies were assessed.

**Results:**

After selection, 48 articles were analyzed. The definition of ultrasound enthesitis and elementary features varied among authors. Grey-scale enthesitis was characterized by increasing thickness (94% of studies), hypoechogenicity (83%), enthesophytes (69%), erosions (67%), calcifications (52%), associated bursitis (46%) and cortical irregularities (29%). Only 46% of studies reported the use of Doppler. High discrepancies were observed on frequency, type of probe and Doppler mode used. Face and content validity were the most frequently evaluated criteria (43%) followed by reliability (29%) and responsiveness (19%).

**Conclusions:**

Ultrasound has evidence to support face, content validity and reliability for the evaluation of enthesitis, though there is a lack of well-reported methodology in most of the studies. Consensus on elementary lesions and standardization of exam is needed to determine the ultrasound definition of enthesitis in grey-scale and in Doppler for future applications.

## Introduction

Enthesitis, that is, the inflammation of insertions of tendons, ligaments and capsules into the bone, is the characteristic sign of ankylosing spondylitis and related pathologies, which are commonly regrouped as spondyloarthritis (SPA). The functioning enthesis dissipates stress over a wide area, including the insertion, immediately adjacent tendon and adjacent bone. The soft tissue components of an enthesis have traditionally been evaluated by clinical examination based on the presence of tenderness and/or swelling while X-rays have been used to assess associated bony changes. The accuracy of these methods, however, is uncertain, which is why new imaging techniques such as ultrasound and magnetic resonance imaging (MRI) have been sought. The role of MRI for assessing the spectrum of pathology in SPA has recently been reported [1,2]. This technique has been most commonly used to assess axial disease. The MRI pattern of SPA enthesitis has been described as a diffuse bone edema adjacent to enthesis, associated with surrounding soft tissue edema [3]. However, MRI lacks sensitivity and specificity for peripheral enthesitis [4]. This can be explained because changes in the fibrous part of the enthesis, where fibroblasts are tightly cross-linked with little scope for accumulation of water, cannot easily be detected with MRI [4,5]. Additionally, MRI cannot easily assess multiple sites or be used to assess the contralateral joints.

Most of the available data on the potential application of ultrasound for rheumatology is currently about the assessment of its role in rheumatoid arthritis with limited data or studies in other rheumatic diseases, among which SPA is themost frequently studied [6-53]. For routine use in daily practice and clinical trials, the assessment of ultrasound performance in terms of metric qualities is recommended [54]. Though several studies have highlighted the value of ultrasound in assessing inflammation of enthesis in SPA, there is no clear agreement on which structures to examine. Even though a clear distinction between the meaning of the word enthesitis and enthesopathy exists in the rheumatologic literature, no clear definition of an enthesitis lesion has been reported in the ultrasound literature. Thus, technical and anatomical issues, combined with a lack of standardization, may have hampered the development and validation of the ultrasound technique applied to clinical practice, or to multicenter studies, in SPA. Consensus definitions for ultrasound-related pathologies were published by the OMERACT (Outcome Measure in Rheumatology in Clinical Trials) ultrasound group in 2005, including enthesopathy [52]. However, no data are available about the implementation of this definition in clinical and research practice.

The objective of this study was to first determine the level of homogeneity in the ultrasound definitions for the principal lesions of enthesitis in the published literature, and second, to evaluate the metric properties of ultrasound for the detection of enthesitis according to the OMERACT filter through a systematic literature review. We focused our review on the anatomical definition of enthesitis, that is, attachment of ligaments or tendons or capsules on bones, which does not imply body tendon nor surrounding tissue, such as bursae.

## Methods

### Search strategy and study selection

The search for original articles concerning humans, published in the English language between January 1985 and May 2010, and referring to peripheral enthesitis and ultrasonography was carried out in PUBMED and EMBASE databases. Reviews or abstracts from scientific congresses were not included.

In order to obtain the largest number of references, the search was performed in two steps in PUBMED with different key words:

- Search 1 was carried out using the following key words « ankylosing spondylitis OR spondylarthropathies OR reactive arthritis OR psoriatic arthritis OR enthesis OR enthesopathy OR rheumatic diseases OR definition » AND « ultrasonography OR ultrasound OR sonography OR Doppler ».

- Search 2 was performed including the key words «entheses OR enthesis OR enthesitis OR enthesopathy ». For both searches key words referred to Mesh Terms or, if not available, to key words present in the title/abstract.

In EMBASE the search was performed with the key words « ankylosing spondylitis OR spondylarthropathy OR reactive arthritis OR Psoriatic arthritis OR Enthesis OR Enthesitis OR Enthesopathy OR Definition » AND « Ultrasonography OR Ultrasound OR Sonography OR Doppler ».

Only references with available abstracts were assessed. Titles, abstracts and full reports of articles identified were systematically screened by one author (FG) with regard to inclusion and exclusion criteria. The final search was verified by a second author (FJ). Articles concerning cadavers were not included in the final selection if they concerned healthy subjects.

Articles which did not meet inclusion criteria were excluded at any step of the study selection.

### Data extraction

All data were extracted from the selected articles using a standardized spreadsheet previously developed and validated for systematic reviews [55,56] . All selected articles were rated in order to determine ultrasound definitions of enthesitis or its characteristics and to evaluate the quality of the studies according to the OMERACT filter [54]. A standardized tool for assessing the quality of the analyzed studies was developed and assessed in a binary mode (yes/no) based on a set of six predefined criteria: 1) Was the recruitment of patients well-defined in the methods section? 2) Was the definition of ultrasound enthesitis clearly defined as well as the definition of each elementary component? 3) Was there a description of ultrasound scanning technique? 4) Was there a description of attempted blinding of observers? 5) Was there a description of enthesitis scoring, and which source was this scoring based on? 6) Was the choice of comparator adequately explained and results completely given? Quality was reported on a scale of 0 to 6, with higher results indicating higher quality.

Particular attention was also given to the definition, quantification and site of detection of Doppler signals, (that is, vascularization detected at enthesis, in the body of the tendon, at cortical bony insertion, in the bursa).

### Evaluation methods

Face and content validity, construct validity, criterion validity and discriminant validity (that is, reliability and responsiveness) were independently evaluated in every paper, including whether the methods for assessing it and their measurement were available or not. Face and content validities, essentially subjective, were analyzed according to the conclusions of authors. Criterion validity was considered achieved when ultrasound results were concurrently or predictively compared with a true "gold standard".

Construct validity was achieved when ultrasound evaluation of enthesitis was demonstrated to be consistent with theoretic concepts (that is, that ultrasound measure of enthesitis is related to other measures of enthesitis).

The evaluation of reliability was divided into two parts: the acquisition phase and reading of images phase. For both we assessed the intra- and inter-observer evaluation. Responsiveness was evaluated by the ability of the tool to demonstrate change, usually in response to an intervention.

### Statistical analysis

Descriptive statistics were used to report data. Frequencies and percentages were used for categorical variables.

## Results

**Figure 1:**
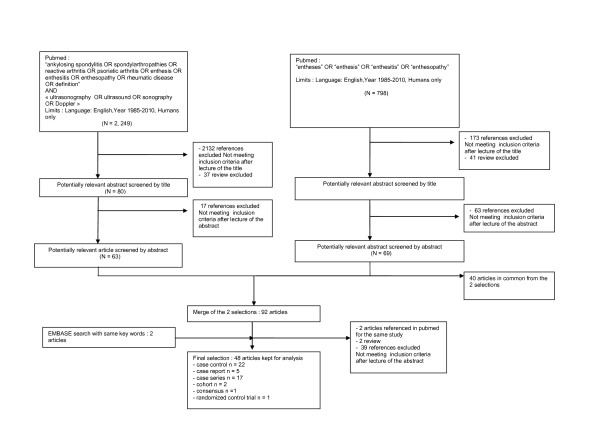
**Flow chart of the articles' selection**.

Figure 1 illustrates the flow chart of the selection of the articles. Of the 3,852 references obtained from databases, 237 abstracts were selected after reading titles, 94 articles were selected after reading abstracts and, finally, 48 articles were analyzed to determine the ultrasonographic enthesitis definition and characteristics. These articles included 22 case-control studies, 5 case-report studies, 17 case-series studies, 2 cohorts, 1 expert consensus and 1 randomized control trial (Table 1). Most of them (n = 37) focused on inflammatory pathologies: spondylarthropathy or ankylosing spondylitis (n = 24), spondylartropathy or other inflammatory rheumatism (n = 3), and psoriatic arthritis (n = 10). Only six studies focused on degenerative involvement of enthesis. Two studies did not report the patients' diagnoses.

**Table 1:** Characteristics of the studies

Year	Authors	Type of Article	Sample Size	Population of interest	Entheses sites of interest	Face validity
**1987**	Maffulli [42]	case series	47	Athletes	A	N
**1989**	Olivieri [47]	case report	1	SPA	A, PF, ischial tuberosity, trochanter	N
**1994**	Lehtinen [40]	case control	39	SPA (ReA, PsA, AS)	pelvic adductor origin, trochanter, ischial tuberosity, PT, A, PF	N
**1995**	Lehtinen [39]	cohort	23	SPA	ischial tuberosity, trochanter, PT, A, PF	N
**1998**	Olivieri [46]	case series	14	SPA	A (hanging free over the edge of the table)	N
**1999**	Gibbon [32]	case control	370	clinically idiopathic plantar fasciitis, SPA, RA, Achilles tendon disease, ankle instability, healthy subjects	A, PF (90°)	N
**2000**	Balint [17]	case report	1	PsA	A (hanging free)	N
**2000**	Galluzzo [29]	case series	31	PsA	A, PF	N
**2001**	Cosentino [19]	RCT	60	patients with talalgia	PF	N
**2002**	Balint [16]	case series	35	SPA (AS, PsA, ReA)	GUESS score: A, PF (90°),PTPI, PTDI, Q (30°)	N
**2002**	D'Agostino [21]	case report	2	SPA	A, PF	N
**2002**	Falsetti [10]	case control	450	SPA ,RA, OA, painful shoulders, healthy subjects	Deltoid tendon insertion	N
**2002**	Falsetti [28]	case control	178	PMR, SPA(PsA, AIBD, uSpA), RA	wrist, elbow, shoulder, hip, knee, ankle, heel	N
**2002**	Frediani [27]	case control	160	PsA, RA, healthy subjects	Q (30°)	N
**2003**	D'Agostino [22]	case control	228	SPA, MBP, RA	A, PF, tibialis anterior tendon, CET, FCT, PT, Q, gluteus medius and minimus tendons	N
**2003**	De Simone [24]	case control	109	psoriatic with arthritis or spondylitis, healthy subjects	A	N
**2003**	Falsetti [11]	case control	598	EOA , NOA , RA, PsA, healthy subjects	A, PF	N
**2003**	Kamel [34]	case series	32	SPA	A, PF	Y
**2004**	Falsetti [9]	case control	157	CCA, OA, healthy subjects	A, PF	N
**2004**	Kamel [35]	case series	16	SPA	PT	N
**2005**	Genc [30]	case control	62	RA , AS, healthy subjects	*	N
**2005**	Ozçakar [48]	case control	50	Psoriasis, healthy subjects	A (Neutral flexion)	N
**2005**	Ozgocmen [49]	case report	1	AS	A	N
**2005**	Wakefield [52]	consensus	-	NA	NA	N
**2006**	Borman [18]	case series	44	SPA (AS, PsA, ReA, uSpA)	A, PF	N
**2006**	Fournie [26]	case control	41	PsA, RA	flexor tendons of the hand	N
**2006**	Kiris [37]	case series	30	AS	MASES **	N
**2006**	Tse [51]	case report	1	AS	A, PF, Q, PT	N
**2007**	Alcalde [14]	case control	54	AS, healthy subjects	A(neutral flexion), PF (neutral flexion),PTPI(60°), PTDI (60°), Q (60°)	N
**2007**	Genc [31]	cohort	54	RA, AS	GUESS *	N
**2007**	Kerimoglu [36]	case series	49	Hemodialysis	GUESS *	N
**2007**	Scarpa [50]	case control	47	PsA , "sine psoriasis"patients	All entheses with increased tracer uptake in scintigraphy	N
**2007**	Wiell [53]	case control	20	PsA, RA, healthy subjects	flexor/extensor tendons of the hands	Y
**2008**	de Miguel [23]	case control	54	SPA(AS, ReA, uSpA, PsA, juvenile SPA), healthy subjects	MASEI score: A (90°), PF (90°), PTPI (70°), PTDI (70°), Q, brachial triceps tendon (90°)	N
**2008**	Filippou [12]	case series	7	Ochronosis	flexor/extensor tendon of the hand, CET, CFT, distal brachial triceps tendon, gluteus medius and minimus tendons, Q, PT, iliotibial band, A, PF, anterior and posterior tibialis tendon, peroneal tendon, toe extensor tendon	N
**2008**	Gisondi [33]	case control	60	Psoriasis, healthy subjects	GUESS *	N
**2008**	Hatemi [7]	case control	160	Behçet, AS, RA, healthy control	GUESS *	Y
**2008**	Klauser [38]	case control	33	SPA (AS, PsA, uSpA, ReA, AIBD), RA, patients with non rheumatic disease	MASES **	N
**2008**	Mc Gonagle [44]	case control	47	SPA(AS, PsA, ReA), healthy subjects	A (90°)	N
**2009**	D'Agostino [20]	case series	5	SPA(AS, uSpA, PsA, AIBD)	Q, PTPI , CET, A, PF	N
**2009**	Filippucci [13]	case series	28	SPA	A (hanging in neutral position)	N
**2009**	Matsos [43]	case series	62	NA	NA	N
**2009**	Munoz-Fernandez [45]	case control	79	SPA, anterior uveitis, healthy subjects	MASEI ***	N
**2009**	Filippucci [25]	case series	NA	PsA	A	N
**2009**	Iagnocco [8]	case series	93	SPA	A(hanging in neutral position)	N
**2010**	Gutierrez [6]	case series	30	PsA	U: A, Apo, PT	N
**2010**	Li [41]	case control	70	SPA(AS, ReA, PsA,AIBD, uSpA), healthy subjects	A (90°)	N
**2010**	Aydin [15]	Case series	43	SPA	A (hanging in neutral position)	N

A, Achilles tendon; AIBD, arthritis associated with inflammatory bowel disease; AS , ankylosing spondylitis; ASIS, anterior superior iliac spines; CET, commun extensor tendon insertion on lateral epicondyle; CFT, common flexor tendon insertion on medial epicondyle; EOA, erosive osteoarthritis; MBP, mechanical low back pain; NA, not available; NOA, nodular osteoarthritis; OA, osteoarthritis; PF, Plantar Fascia; PMR, polymyalgia rheumatica; PsA, psoriatic arthritis; PSIS, posterior superior iliac spines; PT, patellar tendon; PTDI, patellar tendon distal insertion; PTPI, patellar tendon proximal insertion; Q, quadriceps; RA , rheumatoid arthritis; RCT, randomized control trial; ReA, reactive arthritis; SPA, spondylarthropathy; uSpA, undifferenciated spondyloarthritis

* GUESS score: A; PF (90°),PTPI, PTDI, Q (30°), ** MASES: 1^st ^and 7^th ^costosternal joints, PSIS, ASIS, iliac crests, A, 5^th ^lumbar spinous process, *** MASEI score: A (90°), PF (90°), PTPI (70°), PTDI (70°), Q, brachial triceps tendon (90°)

Entheses of lower limbs were the most common studied, especially Achilles tendon (80% of articles) followed by the entheses of upper limbs. No consensus concerning either the location or the number of enthesis to be examined was observed.

### Ultrasound parameters and setting

The description of ultrasound examination was reported in 35 (73%) studies and recommendations on the position of the examined enthesis, especially for lower limbs, were available in most of the studies. Authors predominantly used 90° flexion of the feet during examination of Achilles tendon and Plantar Fascia, 30° to 60° flexion of the knee during examination of the patella ligament and the quadriceps tendon. In more recent studies, a neutral position of the feet was used to perform Achilles tendon entheses examination.

### Definition and description of enthesitis in grey-scale and Doppler modes

In grey-scale a 7.5 MHz or 7.5 to 10 MHz linear probe frequency were used in 15/48 studies while a frequency >10 MHz was used in 23 studies. Information concerning probe characteristics was lacking in four studies.

Table 2 shows definitions or description of ultrasound enthesitis and ultrasound elementary components used for defining enthesitis (for further details see also Table S1 in Additional file 1). Table S2 in Additional file 2 shows ultrasound parameters and equipment used in the different studies. In grey-scale, enthesitis was characterized by the presence of increasing thickness in 45 (94%) studies, hypoechogenicity of the enthesis in 40 (83%) studies, enthesophyte in 33 (69%) studies, erosion in 32 (67%) studies, calcification in 25 (52%) studies, associated with bursitis in 22 (46%) studies or cortical irregularities in 14 (29%) studies. Only 16 (33%) studies described the ultrasound technique of thickness measurement, which was prevalently measured at the point of maximal thickness on the bony insertion (for further details see also Table S3 in Additional file 3).

**Table 2:** Ultrasound definition and description of enthesitis or of its elementary components

**Lear**	**Authors**	**Grey-scale**	**Doppler**	**Definition or description of Enthesitis ♣**	**Elementary components**							
					
					**Echogenicity**	**Thickness**	**Calcific Deposits**	**Enthesophytes**	**Tear**	**Erosions**	**Cortical Irregularities**	**Bursitis**
**1987**	Maffulli [42]	Y	NA	Y	Y	Y	Y	NA	NA	NA	NA	NA
**1989**	Olivieri [47]	Y	NA	NA	NA	Y	NA	NA	NA	NA	NA	NA
**1994**	Lehtinen [40]	Y	NA	Y	U	Y	Y	Y	NA	NA	U	Y
**1995**	Lehtinen [39]	Y	NA	Y	U	Y	Y	Y	NA	NA	U	NA
**1998**	Olivieri [46]	Y	NA	NA	Y	Y	NA	NA	NA	NA	NA	NA
**1999**	Gibbon [32]	Y	NA	Y	Y	Y	Y	Y	NA	Y	NA	NA
**2000**	Balint [17]	Y	Y	NA	NA	Y	NA	NA	NA	NA	NA	Y
**2000**	Galluzzo [29]	Y	NA	Y	Y	Y	Y	NA	NA	NA	NA	NA
**2001**	Cosentino [19]	Y	NA	Y	Y	Y	NA	Y	NA	NA	NA	NA
**2002**	Balint [16]	Y	NA	Y	Y	Y	NA	Y	NA	Y	Y	Y
**2002**	D'Agostino [21]	Y	Y	NA	Y	Y	Y	NA	NA	Y	NA	NA
**2002**	Falsetti [10]	Y	NA	Y	Y	Y	NA	Y	NA	Y	Y	NA
**2002**	Falsetti [28]	Y	NA	Y	Y	Y	NA	Y	NA	Y	NA	NA
**2002**	Frediani [27]	Y	NA	Y	Y	Y	NA	Y	NA	Y	Y	NA
**2003**	D'Agostino [22]	Y	Y	NA	Y	Y	Y	U	U	Y	Y	NA
**2003**	De Simone [24]	Y	NA	NA	Y	Y	Y	NA	Y	NA	NA	Y
**2003**	Falsetti [11]	Y	NA	Y	Y	Y	Y	Y	NA	Y	NA	Y
**2003**	Kamel [34]	Y	NA	Y	Y	Y	Y	Y	Y	NA	NA	NA
**2004**	Falsetti [9]	Y	Y	Y	Y	Y	NA	Y	NA	Y	NA	NA
**2004**	Kamel [35]	Y	NA	Y	Y	Y	Y	NA	NA	NA	NA	Y
**2005**	Genc [30]	Y	NA	Y	Y	Y	NA	Y	NA	Y	NA	Y
**2005**	Ozçakar [48]	Y	NA	NA	U	Y	U	U	U	U	U	Y
**2005**	Ozgocmen [49]	U	Y	NA	NA	U	NA	NA	NA	NA	NA	NA
**2005**	Wakefield [52]	Y	Y	Y	Y	Y	Y	Y	NA	Y	Y	NA
**2006**	Borman [18]	Y	NA	Y	Y	Y	NA	Y	NA	Y	NA	Y
**2006**	Fournie [26]	Y	NA	Y	NA	NA	NA	Y	NA	NA	NA	NA
**2006**	Kiris [37]	Y	Y	NA	Y	Y	Y	Y	U	Y	Y	Y
**2006**	Tse [51]	Y	NA	NA	NA	U	U	U	U	U	U	U
**2007**	Alcalde [14]	Y	NA	Y	Y	Y	Y	NA	Y	Y	NA	Y
**2007**	Genc [31]	Y	NA	Y	Y	Y	NA	Y	NA	Y	NA	Y
**2007**	Kerimoglu [36]	Y	NA	Y	Y	Y	Y	Y	NA	Y	NA	Y
**2007**	Scarpa [50]	Y	Y	Y	Y	Y	Y	Y	NA	Y	Y	NA
**2007**	Wiell [53]	Y	Y	Y	Y	Y	Y	Y	NA	Y	Y	NA
**2008**	de Miguel [23]	Y	Y	NA	Y	Y	Y	Y	NA	Y	NA	Y
**2008**	Filippou [12]	Y	Y	Y	Y	Y	Y	NA	NA	NA	NA	NA
**2008**	Gisondi [33]	Y	NA	Y	Y	Y	NA	Y	NA	Y	NA	Y
**2008**	Hatemi [7]	Y	Y	Y	Y	Y	NA	Y	NA	Y	Y	Y
**2008**	Klauser [38]	Y	Y	Y	Y	Y	NA	Y	NA	Y	NA	NA
**2008**	McGonagle [44]	Y	NA	Y	Y	Y	NA	Y	NA	Y	NA	NA
**2009**	D'Agostino [20]	Y	Y	Y	Y	Y	Y	Y	NA	Y	NA	NA
**2009**	Filippucci [13]	Y	Y	Y	Y	Y	Y	Y	NA	Y	Y	Y
**2009**	Matsos [43]	Y	Y	Y	Y	Y	Y	Y	NA	Y	Y	NA
**2009**	Munoz-Fernandez [45]	Y	Y	NA	Y	Y	Y	Y	NA	Y	NA	Y
**2009**	Filippucci [25]	Y	Y	Y	Y	Y	N	Y	NA	Y	NA	Y
**2009**	Iagnocco [8]	Y	Y	Y	Y	Y	Y	Y	NA	Y	Y	Y
**2010**	Gutierrez [6]	Y	Y	Y	Y	Y	N	Y	NA	Y	NA	Y
**2010**	Li [41]	Y	Y	Y	Y	Y	N	N	NA	Y	Y	NA
**2010**	Aydin [15]	Y	Y	Y	Y	Y	Y	Y	NA	Y	Y	Y

NA, not available, Y, yes

♣: Definition: enthesitis definition reported by the authors, description: enthesitis description or description of enthesitis elementary components, without clear definition reported by the authors

Only 22 out of 48 (46%) studies described the use of Power Doppler to assess enthesitis (Table 3); all of them were published after 2003. Most of the studies took into account the presence of signal Doppler in different locations: tendon, enthesis and bursa. The exact site of measurement of a Doppler signal was described in 12 studies. There were discrepancies regarding the technical recommendations of the use of Doppler with a huge difference of the pulse repetition frequency (PRF) in the studies ranging from 400 Hz to 1,000 Hz.

**Table 3:** Description of enthesitis in Doppler- mode

Year	Authors	Doppler parameters	Description of site of vascularization
2000	Balint [17]	PRF1000 Hz	NA
2002	D'Agostino [21]	PRF 750 Hz, power Doppler gain 50	periosteal bone and enthesis
2003	D'Agostino [22]	PRF 750 Hz, power Doppler gain 50-53dB	cortical bone insertion, body of the tendon, bursa, junction tendon/enthesis
2004	Falsetti [9]	PRF 750-1000 Hz, highest gain level without background noise and low filter	tendon + bursa
2005	Ozgocmen [49]	PRF 0.3-1.5kHz, dynamic range 55dB low wall filter	periosteum and achilles tendon insertion
2005	Wakefield [52]	NA	NA
2006	Kiris [37]	PRF 0.5-1 KHz , dynamic range 50-55dB - low wall filter	tendon + enthesis : no precision concerning the exact location of vascularization
2007	Scarpa [50]	NA	NA
2007	Wiell [53]	PRF 500 Hz	NA
2008	De Miguel [23]	PRF 400Hz, gain 20dB, low wall filter	enthesis, tendon, bursitis
2008	Filippou [12]	NA	NA
2008	Hatemi [7]	PRF 750 Hz	NA
2008	Klauser [38]	8.3 MHz, PRF 500 Hz, low wall filter	NA
2009	D'Agostino [20]	10 MHz, PRF 500 Hz, gain 113 dB	enthesis insertion into the cortical bone
2009	Filippucci [13]	PRF 750 Hz, colour-mode frequency of 9.1 MHz , low wall filters	enthesis, tendon, bursitis
2009	Matsos [43]	NA	U
2009	Munoz-Fernandez [45]	NA	enthesis, tendon, bursitis
2009	Filippucci [25]	NA	U
2009	Iagnocco [8]	PRF 900Hz , Doppler frequency 9.1 MHz, low wall filters	enthesis, tendon, bursitis
2010	Gutierrez [6]	PRF750 Hz , Doppler frequency between 7.5 -14.3 MHz.	U
2010	Li [41]	10 MHz for colour-mode scanning with a focus at 5 mm.	peri-sesamoidal and periosteal areas
2010	Aydin [15]	PRF 750 Hz, colour-mode frequency of 9.1 MHz, low wall filters	enthesis, tendon, bursitis

NA, not available; PRF, pulse repetition frequency; U, unclear; Y, yes,

### Scoring system of enthesitis (grey-scale and Doppler)

Table 4 shows the different ultrasound scoring systems used for evaluating enthesitis. Ultrasound scoring of enthesitis was performed in 20 studies. All of the proposed scoring systems were primarily based on grey scale changes, measuring the thickness of tendon insertion, the presence of erosions, bursitis and enthesophytes. Proposed grading was semi-quantitative in most of them. Only nine studies reported scoring systems of Power Doppler activity of the enthesis, which were generally semi-quantitative [7,8,13,15,20,22,23,37,45], but also quantitative with a proposed cut-off for differentiating between SPA and controls. Five scoring systems were developed at the enthesis level (and mostly concerned Achilles enthesis evaluation), and 15 were developed at the patient level (that is, the scoring system gave information regarding different enthesis sites and allowed the evaluation of global patient inflammatory activity or enthesis structural damage). Two of them, the GUESS (Glasgow Ultrasound Enthesitis Scoring System) score, proposed by Balint *et al*. in 2002 [16] and the SEI (Spanish Enthesitis Index) score, by Alcade *et al*. [14], take into account grey-scale elementary components alone. Both of them are scoring systems developed at the enthesis level and at patient level, and the GUESS was the scoring method most frequently used (7/20).

**Table 4:** Description of enthesitis scoring system

Year	Authors	Enthesis studied	Grey-scale	Doppler mode	Scoring system	Reliability	Sensitivity to change
**2001**	Cosentino [19]	PF	Y	N	grade l: thickening of enthesis (<2 mm thicker than the controlateral asymptomatic side), heterogeneous hypoechogenicity of enthesis and enthesophytosis. grade 2: thickening of enthesis (>2 mm thicker than the controlateral asymptomatic side), heterogeneous hypoechogenicity of enthesis, and enthesophytosis. grade 3: grade 2 with peritendinous oedema.	NA	U
**2002**	Balint [16]	GUESS: A, PF (90°),PTPI, PTDI, Q (30°)	Y	N	GUESS score (0 to 36): Each item scores one point. total possible score on both lower limb is 36 superior pole of the patella- quadriceps tendon enthesis: quadriceps tendon thickness >=6.1mm, suprapatellar bursitis, superior pole of patella erosion, superior pole of patella enthesophyte inferior pole of the patella-proximal patellar ligament enthesis: patellar ligament thickness > = 4 mm, inferior pole of patella erosion, inferior pole of patella enthesophyte tibial tuberosity-distal patellar ligament enthesis: patellar ligament thickness > = 4 mm, infrapatellar bursitis, tibial tuberosity erosion, tibial tuberosity enthesophyte superior pole of the calcaneus-achilles tendon enthesis: Achilles tendon thickness >=5.29 mm, retrocalcaneal bursitis, posterior pole of calcaneus erosion, posterior pole of calcaneus enthesophyte inferior pole of the calcaneus -plantar aponeurosis enthesis: Plantar aponeurosis thickness >=4.4 mm, inferior pole of calcaneus erosion, inferior pole of calcaneus enthesophyte.	U	NA
**2002**	Falsetti [28]	wrist, elbow, shoulder, hip, knee, ankle, calcaneum	Y	N	each item scored according to a semi quantitative score: 1: mild,2: moderate,3:considerable items scored: synovitis, tenosynovitis, enthesitis	U	NA
**2003**	D'Agostino [22]	A, PF, tibialis anterior tendon, CET, CFT, PT, Q, trochanter	Y	Y	stage 1: Vascularization at the cortical junction without abnormal findings in Grey-scale stage 2a: Vascularization associated with swelling and/or decreased echogenicity at the cortical junction in Grey-scale stage 3a: Same as stage 2a, plus erosions of cortical bone and/or calcification of enthesis, and optional surrounding bursitis stage 2b: Abnormal findings in B mode as in stage 2a, but without vascularization stage 3b: Abnormal findings in B mode as in stage 3a, but without vascularization	Y	NA
**2003**	Falsetti [11]	A, PF, retrocalcaneal bursae, subcalcaneal fat pad, cortical bone of posterior and inferior aspects of calcaneum	Y	N	Each inflammatory lesion was graded according to a semi-quantitative scale: grade 1: mild, grade 2: moderate, grade 3: considerable	NA	NA
**2006**	Kiris [37]	MASES *	N	Y	0 = absence, 1 = mild, 2 = moderate, 3 = severe	Y	NA
**2007**	Alcalde [14]	SEI: A, PF (neutral flexion°),PTPI, PTDI, Q (60°)	Y	N	SEI = the total sum of SEI-A and SEI-C. the maximum SEI scoring is 76 points SEI-A (0 to 36): each variable is scored as 0 (absence) or 1 (presence): thickening of tendon/aponeurosis, hypoechogenicity of tendon/aponeurosis, peritendinous/periaponeurotic oedema, bursitis (where applicable) SEI-C (0 to 40): each variable is scored as 0 (absence) or 1 (presence): tendon tear, loss of thickness, tendon calcification, bone erosion.	U	NA
**2008**	De Miguel [23]	MASEI: A (90°), PF (90°), PTPI and PTDI (70°), distal Q tendon, distal brachial triceps tendon (90°)	Y	Y	MASEI score (0 to 136 on both sides): Calcifications were scored on a semi-quantitative score of 0 to 3 Doppler and erosions were scored as 0 or 3 points Scores for tendon structure, tendon thickness and bursa were either 0 or 1. Calcifications were examined at the area of the enthesis insertion, and scored as 0 if absent, or 1 if a small calcification or ossification with an irregularity of enthesis cortical bone profile was seen. Calcifications were given a score of 2 if there was clear presence of enthesophytes or if medium sized calcifications or ossification were observed. Lastly, they were classified as a 3 if large calcifications or ossifications were present. To simplify things, ossifications and enthesophytes at the enthesis were also included as calcifications.	Y	NA
**2008**	Hatemi [7]	GUESS **	Y	Y	GUESS score* + Doppler: one point for each enthese with vascularization. Cumulative score for Doppler (max = 10)	U	NA
**2009**	D'Agostino [20]	Q, PTPI, CET, A, PF	Y	Y	Grey-scale: hypoechogenicity/thickness: 0 to 1, calcification/enthesophyte: 0 to 1, erosion: 0 to 1 Doppler : (0 to 3): 0: no signal, 1: minimal (1 spot), 2: moderate (2 spot), 3: severe (> = 3 spots)or Doppler scored as 0 to 1 (absent-present)	Y	NA
**2008**	Mc Gonagle [44]	A (90°)	Y	N	spur (0 to 3):0 absence, 1: minimal, 2: moderate, 3: large	NA	NA
**2009**	Filippucci [13]	A	Y	Y	soft tissue inflammation (seven items): tendon hypoechogenicity, Entheseal hypoechogenicity, Bursal effusion, PDS signal at tendon level, PDS signal at entheseal level, PDS signal at bursal level tissue damage (five items): Intratendineous calcifications, Entheseal calcifications, Enthesophytes, Bone erosions, Bone irregularities* (not used to calculate total score) (1) a total score for soft tissue inflammation, which resulted from the sum of the scores assigned to the 7 US findings indicative of soft tissue inflammation, ranging from 0 to 7 with presence/absence data and from 0 to 14 with semiquantitative scores; (2) a total score for tissue damage, which resulted from the sum of the scores assigned to the 4 US findings indicative of tissue damage, ranging from 0 to 4 with presence/absence data and from 0 to 8 with semiquantitative scores.	Y	NA
**2009**	Iagnocco [8]	A (neutral position)	Y	Y	All lesions scored on both a dichotomous scale (present/absent) and a 4-point semiquantitative scale (0 = absent, 1 = mild, 2 = moderate, 3 = severe) enthesopathy: tendon hypoechogenicity at the level of bony attachment, tendon thickening at the at the level of bony attachment, intra-tendinous calcifications, enthesophytes, bony erosions, bony cortex irregularities, presence of Doppler signal at the level of bony attachment, presence of intratendinous Doppler signal bursitis: enlargement of deep calcaneal bursa, enlargement of superficial calcaneal bursa tendon lesion: both partial and full-thickness tendon lesions	NA	NA

Y, yes; N, no; U, unclear; NA, not available

A, Achilles tendon; ASIS, anterior superior iliac spines; CET, commun extensor tendon insertion on lateral epicondyle; CFT, common flexor tendon insertion on medial epicondyle; PF, Plantar Fascia; PSIS, posterior superior iliac spines; PT, patellar tendon; PTDI, patellar tendon distal insertion; PTPI, patellar tendon proximal insertion; Q, quadriceps

* MASES: 1^st ^and 7^th ^costosternal joints, PSIS, ASIS, iliac crests, A, 5^th ^lumbar spinous process, ** GUESS score: A, PF (90°),PTPI, PTDI, Q (30°)

Published scoring systems were used both for diagnostic purposes [22,23,53], and for sensitivity to change [15,19,31]. Performance of those scores varied according to the purpose.

### Evaluation of studies according to the OMERACT filter

Table 5 summarizes the characteristics of the 48 selected articles according to the OMERACT filter.

**Table 5:** Summary of reporting according to the OMERACT filter

Year	Authors	Blinded design	Reliability	Construct validity	Criterion validity	Comparator	Responsiveness
**1987**	Maffulli [42]	NA	NA	NA	NA	N	N
**1989**	Olivieri [47]	NA	NA	NA	NA	N	NA
**1994**	Lehtinen [40]	Y	NA	Y	NA	clinical	NA
**1995**	Lehtinen [39]	Y	NA	NA	NA	N	N
**1998**	Olivieri [46]	Y	NA	Y	NA	MRI, clinical	NA
**1999**	Gibbon [32]	N	NA	NA	NA	N	NA
**2000**	Balint [17]	N	NA	NA	NA	N	Y
**2000**	Galluzzo [29]	Y	NA	Y	NA	Xrays, clinical	NA
**2001**	Cosentino [19]	Y	NA	Y	NA	Xrays	Y
**2002**	Balint [16]	Y	U	Y	NA	clinical	NA
**2002**	D'Agostino [21]	NA	NA	NA	NA	N	Y
**2002**	Falsetti [10]	Y	NA	Y	NA	clinical and Xrays	NA
**2002**	Falsetti [28]	Y	Inter	NA	NA	N	NA
**2002**	Frediani [27]	Y	NA	Y	NA	clinical	NA
**2003**	D'Agostino [22]	Y	Intra and inter	Y	NA	clinical	NA
**2003**	De Simone [24]	NA	NA	Y	NA	clinical	NA
**2003**	Falsetti [11]	Y	NA	Y	NA	Xrays	NA
**2003**	Kamel [34]	Y	Intra and inter	Y	NA	MRI	NA
**2004**	Falsetti [9]	Y	NA	Y	NA	clinical	NA
**2004**	Kamel [35]	NA	NA	Y	NA	MRI	NA
**2005**	Genc [30]	Y	NA	NA	NA	N	NA
**2005**	Ozçakar [48]	NA	NA	NA	NA	N	NA
**2005**	Ozgocmen [49]	NA	NA	NA	NA	N	Y
**2005**	Wakefield [52]	NA	NA	NA	NA	N	NA
**2006**	Borman [18]	Y	NA	Y	NA	clinical	NA
**2006**	Fournie [26]	NA	NA	NA	NA	N	NA
**2006**	Kiris [37]	Y	Intra	Y	NA	clinical	NA
**2006**	Tse [51]	NA	NA	Y	NA	MRI	Y
**2007**	Alcalde [14]	Y	Inter	NA	NA	N	NA
**2007**	Genc [31]	Y	NA	NA	NA	N	N
**2007**	Kerimoglu [36]	NA	NA	NA	NA	N	NA
**2007**	Scarpa [50]	Y	NA	NA	NA	N	NA
**2007**	Wiell [53]	Y	Inter	Y	NA	MRI	NA
**2008**	de Miguel [23]	Y	Inter	NA	NA	N	NA
**2008**	Filippou [12]	NA	NA	NA	NA	N	NA
**2008**	Gisondi [33]	Y	U	Y	NA	Xrays	NA
**2008**	Hatemi [7]	Y	Inter	Y	NA	clinical	NA
**2008**	Klauser [38]	Y	NA	Y	NA	clinical	NA
**2008**	Mc Gonagle [44]	Y	NA	NA	Y	histology	NA
**2009**	D'Agostino [20]	Y	Intra and inter	NA	NA	N	NA
**2009**	Filippucci [13]	Y	Inter	NA	NA	N	NA
**2009**	Matsos [43]	NA	NA	NA	NA	N	NA
**2009**	Munoz-Fernandez [45]	Y	U	NA	NA	N	NA
**2009**	Filippucci [25]	NA	NA	NA	NA	N	NA
**2009**	Iagnocco [8]	Y	NA	NA	NA	N	NA
**2010**	Gutierrez [6]	NA	NA	NA	NA	N	NA
**2010**	Li [41]	NA	NA	NA	NA	N	NA
**2010**	Aydin [15]	NA	Intra	NA	NA	N	Y

inter, inter-reliability; intra, intra-reliability; N, no; NA, not available; RCT, randomized control trial; U, unclear; Y, yes

#### Truth

The face, content, criterion and construct validity of ultrasound findings of the enthesis has been tested in only 21 articles (44%). Comparators were clinical examination in 13 studies, MRI in 5 studies, X-ray in 5 studies and histology in 1 study. In three studies, two comparators were used, clinical and X-ray or MRI.

Ultrasound examination was performed blindly from other data in 29 articles (62%).

### Discrimination

#### Reliability

Detailed results of the reliability of the technique, which were evaluated in 14 (29%) studies are only reported in the additional online file (Table S4 in Additional file 4). Among them, eight studies correctly reported the methodology used. Reliability was most frequently tested on static images reading and only two evaluated the acquisition. Only four studies included information on both inter-examiner and intra-examiner reliability. In general, reading reliability was good but acquisition reliability had some deficiencies.

#### Responsiveness

Responsiveness was evaluated in nine studies. Of them, only four included power Doppler evaluation of the enthesis [15,17,21,49] and three used a scoring system [15,19,31]. Ultrasound evaluation of enthesitis was found to be sensitive to change in six studies, whereas three studies did not demonstrate responsiveness, but the evaluation concerned the Grey-scale aspect alone, while in the studies also including Power Doppler the sensitivity to change was greater. Only three articles reported responsiveness regardless of statistical analyses, while six articles were descriptive of changes but did not quantify it.

#### Feasibility

None of the analyzed papers reported information about feasibility of examining entheses using ultrasound.

## Discussion

The present review has demonstrated that ultrasound is considered a valuable tool for assessing enthesitis. Since 1985, when the first description was made by Lehtinen and colleagues, an increasing interest for using this technique in the evaluation of SpA enthesitis has been observed, especially within the last 10 years. This is probably due to the tremendous technological progression of ultrasound equipment. However, standardization of enthesitis assessment by ultrasound would facilitate the dissemination of this technique in daily practice, and also allow adequately trained sonographers to participate in multicenter research studies. A wide variability was observed among studies in the definition of ultrasound enthesitis, associated with a broad heterogeneity of definitions of its elementary components, and the absence of a consensus on technical parameters and methods of examination probably led to the observed heterogeneity in metric properties of the studies according to the OMERACT filter. No consensus concerning either the location or the number of enthesitis to be examined was observed.

Those discrepancies can be explained by the inclusion of studies from 1985 until the present, assuming that ultrasound equipment has improved considerably since that time, and the differences in the quality of equipment may have hampered the detection of those lesions. However, the quality and the attention in the description of enthesitis features have improved in the studies published after 2005, which may be explained by the publication from our group on the preliminary OMERACT definition of enthesopathy [52]. Indeed, previous studies have shown that grey-scale elementary lesions may be observed in both mechanical and inflammatory enthesopathy [11,30]. Yet, in order to help diagnosis, a more specific feature is the detection of inflammatory signs, especially the vascularization.

Since the first observation on the utility of power Doppler for visualizing vascularization of the enthesis as a sign of inflammation made in 2003 [22], an increasing number of studies have included Doppler evaluation. Some authors have well demonstrated the presence of vascularization of the enthesis/bone junction in SPA patients [13,20,23,37]. Even if Doppler use seems to be important, a wide heterogeneity in its use was recorded. Most of the studies referred to the presence of Doppler signal in different locations: tendon, enthesis, bursa. The lack of consensus with regards to the site of examination of abnormal vascularization may contribute to explaining discrepancies among studies. Some authors may call "inflammatory enthesitis" what would be called "tendonitis" by others. Moreover, this review has shown a large difference in the Doppler parameters used among studies. Doppler sensitivity to inflammatory flow (low-velocity flow) depends partly on the settings and partly on the type of equipment.

The differences found in the articles may, therefore, be explained by the lack of consensus on the optimal Doppler settings for enthesitis. Since no information concerning inter-equipment reliability for enthesitis evaluation is available, the different types of ultrasound equipment used may also explain part of the discrepancies observed. Indeed, Doppler sensitivity could have been affected by the type of equipment used; better sensitivity may have been reported with new generation equipment with the highest quality of Doppler parameters.

Only 73% of the studies clearly described acquisition technique. For example, the method for measuring enthesis thickness, which appears as one of the most important features recorded by authors for characterizing enthesitis of the Achilles tendon, was only described in 31% of the studies despite the fact that the necessity of measuring the thickness for defining the presence of enthesitis was reported by 94% of the authors. Measurement methods and site of measurement varied consistently and none of the proposed methods have been extensively tested and validated yet.

The quantification of enthesitis by ultrasound was predominantly performed by using semi-quantitative scoring methods. However, some differences were observed in the evaluation of involvement as all of the proposed scoring systems combined both evaluation of inflammatory activity, mostly by taking into account echogenicity and increased thickness and structural damage, mostly enthesophytes and erosions. As these are all grey-scale changes, this could explain the discrepancy observed in the sensitivity to change. In recent years, there has been more focus on enthesitis vascularization, probably the most interesting and specific feature to differentiate inflammatory enthesitis from mechanical enthesitis [22]. Consequently, enthesitis scoring systems taking Doppler signal into account have been proposed. These scoring systems, taking more into account the inflammatory activity may better present sensitivity to change. Hatemi *et al*. proposed to add a semi-quantitative scoring concerning vascularization to the GUESS score [7].

The proposal of a scoring system validated at the patient level, taking into account inflammatory activity and structural damage is one of the challenges for future studies regardless of ultrasound enthesitis. This implicates to determine which enthesis are the most relevant to include in the scoring system. Moreover, different scoring systems probably would have to be proposed and validated for diagnostic purposes and for monitoring treatment.

Are the analyzed studies correctly designed for applying one or all parameters of validity of the OMERACT filter?

Concerning face validity, most of the authors agreed on the ability of ultrasound to detect enthesitis and related abnormalities. Thus, ultrasound measures of enthesis involvement (both inflammation and structural damage) must be considered to have face and content validity according to the filter. Concerning construct and criterion aspects, validity results are mitigated, probably because of the lack of a good comparator (or reference standard) for evaluating ultrasound enthesitis. In fact, we cannot consider any other imaging techniques, such as X-rays, MRI or clinical evaluation as a true gold standard because they do not measure the same phenomenon. X-rays can only detect structural damage and do not give information concerning soft tissue evaluation, and, therefore, do not give information on inflammatory activity as ultrasounds do. Clinical evaluation underestimates enthesitis involvement due to the difficulty to clearly appreciate the enthesis by physical examination; and a conventional MRI, due to technical limitations, is unable to visualize isolated enthesitis [57]. MRI findings, particularly the measures suggestive of inflammatory activity, need further comparison with ultrasound to evaluate the differences in the imaging techniques and to determine which are the common areas of involvement in order to help further clarification of construct validity. The only real reference which can correctly evaluate ultrasound capabilities is histology, which cannot be currently used because of ethical reasons.

Concerning the discrimination aspect of the filter, published studies have demonstrated that ultrasound can be a reliable and sensitive tool, even if some of the aspects of reliability need to be improved. This applies to the detection of grey-scale abnormalities which were less reliable than the detection of a Doppler signal in the two studies evaluating both the reading and acquisition phases.

Responsiveness was not always evaluated and frequently only a merely description of changes was reported. Among the nine studies in which sensitivity to change was reported, responsiveness was not demonstrated in three which used grey-scale evaluation alone, while all the studies including Doppler evaluation showed responsiveness. Doppler evaluation appeared to be an important feature to take into account in order to evaluate responsiveness to treatment and it should be included in enthesis examination for this purpose. Further evaluation of the responsiveness of enthesitis evaluation should be performed on scoring systems with evidence of statistical difference.

## **Conclusion**

In conclusion, ultrasound enthesitis may be useful for diagnosis or monitoring of SPA patients, but has still to be validated. It appears as a valid (especially for face and content validity) and reliable tool for enthesitis evaluation. A consensus on enthesitis definition is required in order to improve the quality of studies and to improve the value of ultrasound in SPA management. This article is part of the series Advances in the imaging of rheumatic diseases, edited by Mikkel Ostergaard. Other articles in this series can be found at http://arthritis-research.com/series/imaging 

## Abbreviations

GUESS: Glasgow Ultrasound Enthesitis Scoring System; MRI: magnetic resonance imaging; OMERACT: Outcome Measure in Rheumatology in Clinical Trials; PRF: pulse repetition frequency; SEI: Spanish Enthesitis Index; SPA: spondyloarthritis.

## Competing interests

The authors declare that they have no competing interests.

## Authors' contributions

FG performed the literature search, analyzed the data and drafted the manuscript. LT participated in the analysis and interpretation of the literature search and contributed to the manuscript preparation. FJ participated in the literature search and the analysis and interpretation of data. EN and RJW took part in the analysis of data and the manuscript preparation. MADA designed the study, participated in the analysis of data and the preparation of the manuscript. All authors read and approved the final manuscript.

## Supplementary Material

Additional file 1 **Table A: Ultrasound definition and description of enthesitis or of its components**. The table reports an exhaustive description or definition of ultrasound enthesitis reported in the original publications Click here for file

Additional file 2 **Table B: Characteristics of ultrasound parameters and equipments**. The table reports a complete description of the ultrasound equipment and of all parameters (grey-scale and Doppler if present) used in the published studies. Click here for file

Additional file 3 **Table C: Technique of thickness measurement**. The table reports the position of the joint for measuring the enthesis thickness. Click here for file

Additional file 4 **Table D: Intraobserver and interobserver reliability**. The table reports the detailed reliability described into the studies. Click here for file
