# Supplementary material for: Ultrasound in the evaluation of enthesitis: status and perspectives
Source: Arthritis Res Ther. 2011 Nov 17;13(6):R188. doi: 10.1186/ar3516 (PMC3334637; doi:10.1186/ar3516)
Supplement: Additional file 1 — Table A: Ultrasound definition and description of enthesitis or of its components. The table reports an exhaustive description or definition of ultrasound enthesitis reported in the original publications [file ar3516-S1.DOC]

**Additional files**

**Table S1: Ultrasound definition and description of enthesitis or of its components.**

| Year | Authors | Definition of Enthesitis | Description of Enthesis Abnormalities | Definition or description reported in the original publication |
| --- | --- | --- | --- | --- |
| 1987 | Maffulli [42] | NA | Y | Description: enlargement of the distal part of the tendon, with alterations of the normal pattern of echogenicity |
| 1989 | Olivieri [47] | NA | NA | NA |
| 1994 | Lehtinen [40] | Y | NA | Definition: US features considered to be signs of enthesitis were thickening in the tendon insertion, intratendinous focal changes, calcitic deposits in the insertions, and periosteal changes. |
| 1995 | Lehtinen [39] | Y | NA | * |
| 1998 | Olivieri [46] | NA | NA | NA |
| 1999 | Gibbon [32] | NA | Y | description of plantar fasciitis: thickness increased, abnormal echogenicity, peritendinous fluid, calcifications, calcaneal spurs, erosions |
| 2000 | Balint [17] | NA | NA | NA |
| 2000 | Galluzzo [29] | Y | NA | * |
| 2001 | Cosentino [19] | Y | NA | Definition: presence of enthesophytosis, heterogeneous hypoechogenicity, swelling of enthesitis, peritendinous oedema |
| 2002 | Balint [16] | Y | NA | Definitions of ultrasound signs of enthesitis: thickened enthesis, fluid collection, erosion, bony spur  Definition of Bony erosion: a cortical breakage with a step down contour defect  Definition of enthesophyte: a step up of bony prominence at the end of the normal bone contour  Definition of bursitis: well circumscribed localised anechoic or hypoechoic area at the site of an anatomical bursa and which was compressible by the transducer. Normal bursa dimension <2mm in the short axis. |
| 2002 | D’Agostino [21] | NA | NA | NA |
| 2002 | Falsetti [10] | Y | NA | Definition of deltoid enthesitis: localized thickening of the enthesis, heteregenous hypoechogenicity and swelling of the enthesitis associated or not with ireegularities of the anterolateral aspect of the acromion (subchondral rearrangement and initial enthesophyte) |
| 2002 | Falsetti [28] | Y | NA | Definition: heterogeneous hypoechogenicity and thickening of enthesis, possibly associated with enthesophyte, erosion and peritendinous oedema |
| 2002 | Frediani [27] | Y | NA | Definition: heterogeneous hypoechogenicity and thickening of the enthesis, gross irregularity of the patella (more than 2 mm), enthesophyte of more than 5 mm, erosions. At least one of this criteria was necessary for the diagnosis of enthesitis |
| 2003 | D’Agostino [22] | NA | NA | NA |
| 2003 | De Simone [24] | NA | NA | NA |
| 2003 | Falsetti [11] | Y | NA | Definition of enthesitis: heterogeneous hypoechogenicity and thickening of enthesis possibly associated with enthesophyte, erosions, peritendinous oedema.  Definition of Plantar fasciitis:  heterogeneous hypoechogenicity and thickening of enthesis (> 4mm) possibly associated with enthesophytosis, erosions and perifascial oedema. |
| 2003 | Kamel [34] | NA | Y | Description of enthesis abnormalities:loss of the normal fibrillar pattern, blurring of the tendon margins, irregular fusiform thickening and intra-tendinous lesions with ill defined focal tendons defects filled with a mixture of fluid, fat and/or granulation tissue, with loss of their tightly packed echogenic dots. US examination detected early calicification foci. |
| 2004 | Falsetti [9] | Y | NA | Definition of Achilles enthesopathy: heterogeneous hypoechogenicity, thickening of the insertional tract of the tendon (>5.9 mm at the postero-superior calcaneal surface), sometimes associated with enthesophytosis, erosions, peritendinous oedema |
| 2004 | Kamel [35] | NA | Y | Description of enthesis abnormalities:loss of the normal fibrillar echo texture, no homogeneous pattern, blurring of the patella tendon margins, irregular focal or generalised increased tendon thickness, focal ill defined tendon defects with loss of their tightly packed echogenic dots |
| 2005 | Genc [30] | Y | NA | ** |
| 2005 | Ozçakar [48] | NA | NA | NA |
| 2005 | Ozgocmen [49] | NA | NA | NA |
| 2005 | Wakefield [52] | Y | NA | OMERACT Definition of Enthesopathy: abnormally hypoechoic (loss of normal fibrillar architecture) and/or thickened tendon or ligament at its bony attachment (may occasionally contain hyperechoic foci consistent with calcification) seen in 2 perpendicular planes that may exhibit Doppler signal and/or bony changes including enthesophytes, erosions, irregularities. |
| 2006 | Borman [18] | Y | NA | ** |
| 2006 | Fournie [26] | NA | Y | Description of enthesitis: abnormalities denoting enthesitis included juxtaarticular periosteal reaction, capsular enthesophytes, and enthesopathy at the attachment of the deep flexor tendon on the distal phalanx |
| 2006 | Kiris [37] | NA | NA | NA |
| 2006 | Tse [51] | NA | NA | NA |
| 2007 | Alcalde [14] | NA | Y | Description of enthesitis: acute inflammation : increase of thickness, hypoechogenicity, peritendinous oedema, bursitis  chronic lesions : tears, loss of thickness, intratendinous calcification, bone erosion |
| 2007 | Genc[31] | **Y** | NA | ** |
| 2007 | Kerimoglu [36] | Y | NA | ** |
| 2007 | Scarpa [50] | Y | NA | *** |
| 2007 | Wiell [53] | Y | NA | Definition: intratendinous hypoechoic,enlargement and/or intratendinous hyperechoic bands with or without acoustic shadow and/or periosteal irregularities and/or intratendinous power Doppler signal at the enthesis |
| 2008 | De Miguel [23] | NA | NA | MASEI score : no definition of enthesitis but definition of some of its components  Examination of enthesis includes: presence of calcifications/enthesophytes, bursitis, bony erosion, fascia and tendon abnormal thickness  Definition of enthesophytes: hyperochoic spurs forming at a tendon insertion into bone, growing in the direction of the natural pull of the tendon involved. Ossifications and enthesophytes at the enthesis were also included as calcifications.  Definition of bony erosion: a cortical breakage with a step down bone contour defect in longitudinal and transversal axes.  Definition of bursitis: well circumscribed, localized anechoic or hypoechoic area at the site of an anatomical bursa, which is compressible by the transducer. |
| 2008 | Filippou [12] | NA | Y | Description: presence of hyperechoic deposits without acoustic shadow, thickening, hypoechogenicity, Doppler (acute) |
| 2008 | Gisondi [33] | Y | NA | ** |
| 2008 | Hatemi [7] | Y | NA | ** |
| 2008 | Klauser [38] | Y | NA | Definition of Chronic enthesitis: In grey-scale, extra-articular cortical irregularities including erosions (defined as cortical breakage with a step down contour defect within the areas where tendons, ligaments or joint capsules attach to bone) with or without new bone fonnation (step up bony prominence at the end of the normal bone contour), and local hypoechoic swelling of the entheses (indicated by a convex surface of the enthesis adjacent to bone) without signs of vascularization were defined as sonographic signs of chronic enthesitis.  Definition of Acute enthesitis: blood flow was examined at the enthesis using power Doppler mode. Detection of vascularization was defined as a sign of acute enthesitis |
| 2008 | McGonagle [44] | NA | Y | Description : hypoechoic thickening (Acute enthesitis), loss of normal fibrillar architecture |
| 2009 | D’Agostino [20] | Y | NA | Definition: any of these lesions was considered a feature of enthesitis:  - morphologic abnormalities : increased thickness and/or hypoechogenicity of enthesis insertion  - structural abnormalities: calcifications and/or enthesophytes, erosions  - vascularisation at the enthesis insertion into the cortical bone as an inflammatory sign |
| 2009 | Filippucci [13] | Y | Y | *** |
| 2009 | Matsos [43] | Y | NA | *** |
| 2009 | Munoz-Fernandez [45] | NA | NA | **** |
| 2009 | Filippucci [25] | Y | NA | Definition: hypoechoic swelling of the tendon insertion, increase of blood flow detectable with power Doppler and retrocalcaneal bursal enlargement. Ultrasound changes at the bony level include erosions and enthesophytes. |
| 2009 | Iagnocco [8] | Y | NA | *** with more precisions concerning the location of abnormalities:tendon hypoechogenicity at the level of bony attachment, tendon thickening at the at the level of bony attachment, intra-tendinous calcifications, enthesophytes, bony erosions, bony cortex irregularities, presence of Doppler signal at the level of bony attachment, presence of intratendinous Doppler signal |
| 2010 | Gutierrez [6] | NA | Y | Description of enthesis abnormalities:  Early enthesopathy: entheseal thickening, hypoechogenicity, and fibrillar separation due to intratendineous oedema, with or without associated bursitis and different patterns of power Doppler signal distribution.  In the late stages: bony cortex changes may be related to the presence of enthesophytes and/or bone erosions |
| 2010 | Li [41] | Y | NA | Definition : lack of fibrillar echotexture of the tendon with/without focal and irregular thickening of the sesamoid-periosteal fibrocartilage , enthesis tendon swelling with a recognizable difference compared with the contralateral enthesis, cortical bone irregularity in the areas of the enthesis fibrocartilage , cortical bone erosions in the areas of the periosteal and enthesis fibrocartilage , various degrees of abnormal vascularizations in the peri-sesamoidal and periosteal areas |
| 2010 | Aydin [15] | Y | Y | *** |

NA: not available, Y: yes

*: Definition described by Lehtinen and al : US features considered to be signs of enthesitis were thickening in the tendon insertion, intratendinous focal changes, calcitic deposits in the insertions, and periosteal changes.

**: Definition described by Balint and al. for the GUESS score: Definitions of ultrasound signs of enthesitis: thickened enthesis, fluid collection, erosion, bony spur. Definition of Bony erosion: a cortical breakage with a step down contour defect. Definition of enthesophyte: a step up of bony prominence at the end of the normal bone contour. Definition of bursitis: well circumscribed localised anechoic or hypoechoic area at the site of an anatomical bursa and which was compressible by the transducer. Normal bursa dimension <2mm in the short axis.

***: OMERACT Definition described by wakefield and al. : OMERACT Definition of Enthesopathy: abnormally hypoechoic (loss of normal fibrillar architecture) and/or thickened tendon or ligament at its bony attachment (may occasionally contain hyperechoic foci consistent with calcification) seen in 2 perpendicular planes that may exhibit Doppler signal and/or bony changes including enthesophytes, erosions, irregularities.

****: MASEI score : no definition of enthesitis but definition of some of its components by De Miguel and al. examination of enthesis includes: presence of calcifications/enthesophytes, bursitis, bony erosion, fascia and tendon abnormal thickness. Definition of enthesophytes: hyperochoic spurs forming at a tendon insertion into bone, growing in the direction of the natural pull of the tendon involved. Ossifications and enthesophytes at the enthesis were also included as calcifications. Definition of bony erosion: a cortical breakage with a step down bone contour defect in longitudinal and transversal axes. Definition of bursitis: well circumscribed, localized anechoic or hypoechoic area
