# Supplementary material for: Ultrasound in the evaluation of enthesitis: status and perspectives
Source: Arthritis Res Ther. 2011 Nov 17;13(6):R188. doi: 10.1186/ar3516 (PMC3334637; doi:10.1186/ar3516)
Supplement: Additional file 2 — Table B: Characteristics of ultrasound parameters and equipments. The table reports a complete description of the ultrasound equipment and of all parameters (grey-scale and Doppler if present) used in the published studies. [file ar3516-S2.DOC]

**Additional files**

**Table S2: Characteristics of ultrasound parameters and equipments**

| Year of publication | Authors | Readers | US equipment | Probe | Doppler parameters |
| --- | --- | --- | --- | --- | --- |
| 1987 | Maffulli [42] | 2 | na | na | _ |
| 1989 | Olivieri [47] | u | ansaldo | na | _ |
| 1994 | Lehtinen [40] | u | US acuson 128 | 7,5 MHz | _ |
| 1995 | Lehtinen [39] | u | US acuson 128 | 7,5 MHz | _ |
| 1998 | Olivieri [46] | 1 | esaote AU450 | 7,5 MHz | _ |
| 1999 | Gibbon [32] | 1 | ATL HDI 3000 | 10-15 MHz | _ |
| 2000 | Balint [17] | u | ATL HDI 3000 | 10-5 MHz | PRF1000 Hz |
| 2000 | Galluzzo [29] | 1 | Hitachi AU 280 | 7.5 MHz | _ |
| 2001 | Cosentino [19] | 1 | toshiba tosbee SAL 240 | 7,5 MHz | _ |
| 2002 | Balint [16] | 1 | ATL HDI 3000 | 7-14 MHz | _ |
| 2002 | D’Agostino [21] | u | esaote AU harmonic | 13 MHz | PRF 750 Hz, power Doppler gain 50 |
| 2002 | Falsetti [10] | 1 | toshiba tosbee SAL 240 | 7,5 MHz | _ |
| 2002 | Falsetti [28] | 2 | toshiba tosbee SAL 240 | 7,5 MHz | _ |
| 2002 | Frediani [27] | 1 | toshiba SAL 240 | 7,5 MHz | _ |
| 2003 | D’Agostino [22] | 1 | esaote AUS | 13 Mhz | PRF 750 Hz, power Doppler gain 50-53dB |
| 2003 | De Simone [24] | u | sonolayer SSA - 250 tohiba | 7,5 MHz | _ |
| 2003 | Falsetti [11] | 1 | toshiba tosbee SAL 240 | 7,5 Mhz | _ |
| 2003 | Kamel [34] | 1 | HDI 3000 ATL US | 12Mhz | _ |
| 2004 | Falsetti [9] | 1 | esaote technos | 10-13 MHz | PRF 750-1000 Hz, highest gain level without background noise and low filter |
| 2004 | Kamel [35] | u | HDI 3000 ATL US | 12 MHz | _ |
| 2005 | Genc [30] | 1 | siemens sonoline | 7,5 Mhz | _ |
| 2005 | Ozçakar [48] | u | Hitachi | 7.5 MHz | _ |
| 2005 | Ozgocmen [49] | u | toshiba aphio SSA 770 | 7-13 MHz | PRF 0.3-1.5kHz, dynamic range 55dB low wall filter |
| 2005 | Wakefield [52] | na | na | na | na |
| 2006 | Borman [18] | 1 | Hitachi EUB 555 | 7.5-10 MHz | _ |
| 2006 | Fournie [26] | 1 | siemens sonoline | 13.5 MHz | _ |
| 2006 | Kiris [37] | 1 | toshiba | 7-14 MHz | PRF 0.5-1 KHz , dynamic range 50-55dB - low wall filter |
| 2006 | Tse [51] | u | sequoia system | 8-15 Mhz | _ |
| 2007 | Alcalde [14] | 2 readers | toshiba | 7,5 Mhz | _ |
| 2007 | Genc[31] | 1 | siemens sanoline | 7,5 Mhz | _ |
| 2007 | Kerimoglu [36] | 1 radiologist | siemens | 5-13 Mhz | _ |
| 2007 | Scarpa [50] | 1 | philips iU22 | na | na |
| 2007 | Wiell [53] | 2 | GE logic 9 U US system | 9-14 MHz | PRF 500 Hz |
| 2008 | De Miguel [23] | 1 | GE logic 5 pro US system | 7-12 MHz | PRF 400Hz, gain 20dB, low wall filter |
| 2008 | Filippou [12] | 1 | esaote technos | 7.5-13 MHz | NA |
| 2008 | Gisondi [33] | 1 radiologist+ 1 rheumatologist | ATL HDI 3000 | 10-15 MHz | _ |
| 2008 | Hatemi [7] | 2 | shimadzu 1200 | 8-15 MHz | PRF 750 Hz |
| 2008 | Klauser [38] | 1 | esaote technos | 10-13 MHz | 8.3 MHz, PRF 500 Hz, low wall filter |
| 2008 | McGonagle [44] | 2 | phillips HDI 5000 and ESAOTE technos | 15-8 MHz and 14-10 MHz |  |
| 2009 | D’Agostino [20] | 5 | esaote technos | 13 MHz | 10 MHz, PRF 500 Hz, gain 113 dB |
| 2009 | Filippucci [13] | 3 | MyLab 70 XVG Esaote | 6–18 MHz linear probe | PRF 750 Hz, colour-mode frequency of 9.1 MHz , low wall filters |
| 2009 | Matsos [43] | na | Toshiba and Logic9 GE | 5-12 Mhz | NA |
| 2009 | Munoz-Fernandez [45] | 2 | GE Logiq 5 Pro US system | 5–12 MHz | NA |
| 2009 | Filippucci [25] | na | MyLab70 XVG system Esaote | 6–18 MHz and 4–13 MHz broadband linear probes. | NA |
| 2009 | Iagnocco [8] | na | logiq9 (GE) | 8-15Mhz | PRF 900Hz , Doppler frequency 9.1 MHz, low wall filters |
| 2010 | Gutierrez [6] | 2 | MY Lab 70 ESAOTE and Technos ESAOTE | mylab70:6–18 MHz , Technos:8–14 MHz | PRF750 Hz , Doppler frequency between 7.5 -14.3 MHz. |
| 2010 | Li [41] | 1 | ACUSON Sequoia 512 | 15L8w transducer | 10 MHz for colour-mode scanning with a focus at 5 mm. |
| 2010 | Aydin [15] | 2 | MyLab 70 Esaote | 6–18MHz | PRF 750 Hz, colour-mode frequency of 9.1 MHz, low wall filters |

(U = unclear, NA= not available)
