# Supplementary material for: Ultrasound in the evaluation of enthesitis: status and perspectives
Source: Arthritis Res Ther. 2011 Nov 17;13(6):R188. doi: 10.1186/ar3516 (PMC3334637; doi:10.1186/ar3516)
Supplement: Additional file 3 — Table C: Technique of thickness measurement. The table reports the position of the joint for measuring the enthesis thickness. [file ar3516-S3.DOC]

**Additional files**

**Table S3: Technique of thickness measurement**

| Year | Authors | Ultrasound technique of thickness measurement |
| --- | --- | --- |
| 1998 | Olivieri [46] | Neutral position of the feet. Sagittal scan at the superior calcaneal surface and 3 cm above |
| 1999 | Gibbon [32] | 3 measures where the plantar fascia crosses the anterior aspect of the inferior border of the calcaneus |
| 2002 | Balint [16] | Ligament, aponevrosis or tendon at the point of maximal thickness proximal to the bony insertion |
| 2003 | de Simone [24] | Middle third of the tendon |
| 2004 | Falsetti [9] | Measurement of the insertional tract of the achilles tendon. Thickening : measure >5.9 mm |
| 2005 | Genc [30] | U * |
| 2005 | Ozçakar [48] | Sagittal, insertion on the bone |
| 2006 | Borman[18] | U * |
| 2007 | Genc [31] | U * |
| 2007 | Kerimoglu [36] | Tendon near insertion to the bone |
| 2008 | De Miguel [23] | Measure at the point of maximal thickness on the bony insertion |
| 2008 | Gisondi [33] | U * |
| 2008 | Hatemi [7] | Ligament, aponevrosis or tendon at the point of maximal thickness proximal to the bony insertion. Cut-off determined by adding 2 SD to the means values for healthy subjects. Q: >6.1, patellar: >4, A:>5.3, plantar fascia>4.4 mm |
| 2009 | Filippucci [13] | Achilles tendon thickness was measured by placing the calipers on the tendon margins at the insertion of the deeper tendon margin into the calcaneal bone. The threshold value for the identification of Achilles tendon thickening was 5.29 mm according to Balint et al |
| 2009 | Munoz-Fernandez [45] | Measure at the point of maximal thickness on the bony insertion |

* GUESS: Ligament, aponevrosis or tendon at the point of maximal thickness proximal to the bony insertion
