# Supplementary material for: Ultrasound in the evaluation of enthesitis: status and perspectives
Source: Arthritis Res Ther. 2011 Nov 17;13(6):R188. doi: 10.1186/ar3516 (PMC3334637; doi:10.1186/ar3516)
Supplement: Additional file 4 — Table D: Intraobserver and interobserver reliability. The table reports the detailed reliability described into the studies. [file ar3516-S4.DOC]

**Additional files**

**Table S4 : Intraobserver and interobserver reliability** (K= kappa, ICC= intraclass correlation coefficient, U = unclear, Y=yes, NA= not available)

| Year | Authors | Intra observer | Reading | Acquisition | Interobserver | Reading | Acquisition | Design satisfactory | Blindly |
| --- | --- | --- | --- | --- | --- | --- | --- | --- | --- |
| 2002 | Balint [16] | k=0.9 | U | U | NA | NA | NA | N | U |
| 2002 | Falsetti [28] | NA | NA | NA | k=0.92 | U | U | U | U |
| 2003 | D'Agostino [22] | Y | k=0,97 | NA | Y | k=0,83 | NA | Y | Y |
| 2003 | Kamel [34] | NA | NA | NA | Y | 0,72 | NA | Y | Y |
| 2006 | Kiris [37] | Y | k= 0.82 | NA | NA | NA | NA | N | Y |
| 2007 | Alcalde [14] | NA | NA | NA | alpha=0,84,  ICC: 0,72 (0,56-0,83) | U | U | U | U |
| 2007 | Wiell [53] | NA | NA | NA | Y | NA | k: na, 83- 100% agreement | Y | Y |
| 2008 | de Miguel [23] | NA | NA | NA | ICC 0.6 - 0.86 | ICC : 0.6 | ICC : 0.86 | Y | Y |
| 2008 | Gisondi [33] | k=0,9 tout site,  k=0,91 thickness,  k=0,98 erosion,  k=0,98 bursitis,  k=0,88 enthesophyte | U | U | NA | NA | NA | N | Y |
| 2008 | Hatemi [7] | NA | NA | NA | Y | NA | k: 0.55  ICC: 0.71 | Y | Y |
| 2009 | D'Agostino [20] | Y | Step1:  Doppler (0-3) : k=0.83-0.96  Doppler (0-1) : k=1  Morphologic(0-1) :k=0.3-0.89  Calcifications/enthesophytes (0-1) : k=-0.05-0.48  Erosions (0-1) : k=0.73-0.76 | Step 2:  Doppler (0-3) : k=0.9-1  Doppler (0-1) : k=0.9-1  Morphologic(0-1) : k=0.47-1  Calcifications/enthesophytes (0-1) : k=-0.14-0.4  Erosions (0-1) : k=0.25-1  Step 3:  Doppler (0-3) : k=0.32-0.88  Doppler (0-1) : k=0.46-1  Morphologic(0-1) : k=0.48-0.63  Calcifications/enthesophytes(0-1) : k=0.21-0.70  Erosions (0-1) : k=0.3-0.8 | Y | Step1:  Doppler (0-3) : k=0.59  Doppler (0-1) : k=0.97  Morphologic(0-1) : k=0.38  Calcifications/enthesophytes (0-1) : k=0.09  Erosions (0-1) : k=0.81 | Step 2:  Doppler (0-3) : k=0.51  Doppler (0-1) : k=0.65  Morphologic(0-1) : k=15  Calcifications/enthesophytes (0-1) : k=0.25  Erosions (0-1) : k=0.33  Step 3:  Doppler (0-3) : k=0.57  Doppler (0-1) : k=0.58  Morphologic(0-1) : k=0.41  Calcifications/enthesophytes (0-1) : k=0.30  Erosions (0-1) : k=0.24 | Y | Y |
| 2009 | Filippucci [13] | Y | 0.462 to 1.000 | NA | Y | NA | unweighted k 0.497  to 0.913 | Y | Y |
| 2009 | Munoz-Fernandez [45] | ICCs: 0.89 (95% CI 0.64–0.97) and 0.94 (95% CI 0.82–0.98) for the 2 ultrasonographers. | U | U | NA | NA | NA | U | U |
| 10 | Aydin [15] | Y | k = 0.816 | NA | k = 0.647 | U | U | U | U |
